# Altered (neo-) lacto series glycolipid biosynthesis impairs α2-6 sialylation on *N*-glycoproteins in ovarian cancer cells

**DOI:** 10.1038/srep45367

**Published:** 2017-03-30

**Authors:** Shahidul Alam, Merrina Anugraham, Yen-Lin Huang, Reto S. Kohler, Timm Hettich, Katharina Winkelbach, Yasmin Grether, Mónica Núñez López, Nailia Khasbiullina, Nicolai V. Bovin, Götz Schlotterbeck, Francis Jacob

**Affiliations:** 1 Ovarian Cancer Research, Department of Biomedicine, University Hospital Basel, University of Basel, Basel, 4031, Switzerland; 2 Glyco-oncology, Ovarian Cancer Research, Department of Biomedicine, University Hospital Basel, University of Basel, Basel, 4031, Switzerland; 3 School of Life Sciences, University of Applied Sciences and Arts Northwestern Switzerland, Muttenz, 4132, Switzerland; 4 Shemyakin- Ovchinnikov Institute of Bioorganic Chemistry, Russian Academy of Sciences, Moscow, 117997, Russian Federation

## Abstract

The (neo-) lacto series glycosphingolipids (nsGSLs) comprise of glycan epitopes that are present as blood group antigens, act as primary receptors for human pathogens and are also increasingly associated with malignant diseases. Beta-1, 3-*N*-acetyl-glucosaminyl-transferase 5 (B3GNT5) is suggested as the key glycosyltransferase for the biosynthesis of nsGSLs. In this study, we investigated the impact of CRISPR-*Cas9* -mediated gene disruption of *B3GNT5* (∆*B3GNT5*) on the expression of glycosphingolipids and *N*-glycoproteins by utilizing immunostaining and glycomics-based PGC-UHPLC-ESI-QTOF-MS/MS profiling. ∆*B3GNT5* cells lost nsGSL expression coinciding with reduction of α2-6 sialylation on *N*-glycoproteins. In contrast, disruption of *B4GALNT1*, a glycosyltransferase for ganglio series GSLs did not affect α2-6 sialylation on *N*-glycoproteins. We further profiled all known α2-6 sialyltransferase-encoding genes and showed that the loss of α2-6 sialylation is due to silencing of *ST6GAL1* expression in ∆*B3GNT5* cells. These results demonstrate that nsGSLs are part of a complex network affecting *N*-glycosylation in ovarian cancer cells.

Glycosphingolipids (GSLs) have been shown to be essential in a wide variety of biological events - such as cell signalling, modification of insulin and EGF-receptor activities, and modulation of Notch ligand activity in *Drosophila*^1^. They also interact with well-known proteins such as EGFR^2^, TGFβ1R^3^, and VEGFR^4^ in various malignancies. GSLs are usually divided into two major families, known as galactosylated or glucosylated ceramides. The latter (glucosylceramide-related glycosphingolipids) is further divided into three major classes based on the action of specific glycosyltransferases; globo- (A4GALT), ganglio- (B4GALNT1 and ST3GAL5), and (neo-) lacto (B3GNT5) -series. Additional elongation of lipid-linked carbohydrate chains is determined by the intracellular localization, which is usually embedded in the endomembrane system and the regulation of specific glycosyltransferases within the GSL-glycan biosynthetic pathway^5^.

The *B3GNT5* gene encodes the glycosyltransferase β-1,3-*N*-acetylglucosaminyl transferase 5, which attaches *N*-acetylglucosamine (GlcNAc) to lactosylceramide (Galβ1-4Glcβ1-1Ceramide) resulting in the precursor lactotriaosylceramide (Lc3, GlcNAcβ1-3Galβ1-4Glcβ1-1Ceramide) for synthesis of lacto (Type 1) and neolacto-series (Type 2) GSLs^6^ (Fig. 1A). This enzyme, together with its associated glycosidic product (Lc3), plays a role in human malignant diseases, embryonic development and cell differentiation. Specifically, Lc3 was shown to be elevated on the cell surface of human pro-myelocytic leukemia HL60 cells^6^,^7^. Moreover, it has also been suggested as a differentiation-associated GSL in the bone marrow of acute myeloid leukemia patients with corresponding elevated *B3GNT5* expression^8^. In mice experiments, the current data on the potential function of B3GNT5 seems rather controversial, possibly due to the use of a multicellular organism and different mutation-generating techniques, thereby limiting biological interpretations regarding its role^9^,^10^,^11^. Other than Lc3, it is also known that B3GNT5 is the key enzyme for several GSL structures associated with human diseases such as sialyl-Lewis x^12^ and human blood groups ABO and P [based on the Kyoto Encyclopedia of Genes and Genomes (KEGG, http://www.genome.jp/kegg/)]. Nevertheless, the biological function of B3GNT5-mediated GSLs is rather limited in the context of cancer and remains to be explained.

In this study, we successfully created a site-specific and heritable *B3GNT5* knockout in human cancer cell lines. By utilizing the CRISPR *Cas9* technology, we established an experimental tool for studying the function of B3GNT5-mediated GSLs, namely the entire (neo-) lacto series (nsGSL). In addition, we also performed a glycomics profiling using mass spectrometry to evaluate the effects of this GSL gene knockout on the entire glycome repertoire of membrane proteins and lipids. The specific glycan alterations described in this study are consistent in two ovarian cancer cell lines and seem to be specific for B3GNT5. It is envisioned that this gene-editing technology will serve as a useful platform to facilitate the downstream investigation of B3GNT5 and its regulation of both GSL and protein glycosylation in cancer development and progression.

## Results

### (Neo-) lacto- series glycosphingolipids are expressed on cancer cells

**Figure 1:**
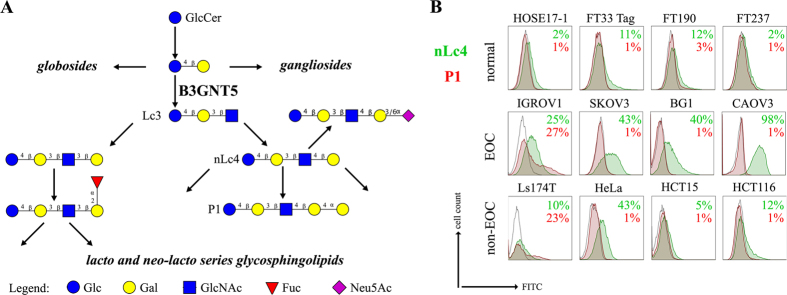
The heterogeneous expression of (neo-) lacto series glycosphingolipids on normal and cancer cell lines. (**A**) Depiction of the three major glycosphingolipid series - globo, ganglio, and (neo-) lacto series glycosphingolipids. B3GNT5 attaches GlcNAc to the LacCer synthesizing Lc3, the precursor of all nsGSLs. Glycosidic linkages are displayed next to CFG notated monosaccharides. (**B**) Representative histograms of flow cytometry data on normal and cancer cell lines stained for paragloboside (nLc4, green) and P_1_ (red). Grey histogram depicts the negative control. Values within each plot show the mean out of three independent experiments for nLc4 (green) and P_1_ (red).

As part of our initiative to comprehensively characterize nsGSLs, we have recently reported the presence of paragloboside (nLc4, precursor of P_1_) and P_1_ pentasaccharide in tumor specimens and immortal ovarian cancer cells using two complementary methods; PGC-LC-ESI-MS/MS and flow cytometry^13^,^14^,^15^. In this study, we extended the profiling of nsGSLs into three distinct groups; Normal (HOSE17-1, FT33-Tag, FT190 and FT237 which were suggested as a potential origin of epithelial ovarian cancer^16^,^17^), Ovarian (IGROV1, SKOV3, BG1, and CAOV3), and Non-ovarian cancer cell lines (Ls174T, HeLa, HCT15, and HCT116). The flow cytometry data revealed a generally lower expression of nsGSLs in normal cells (nLc4 2–12% and P_1_ 1–3%), while all four of the ovarian cancer cell lines displayed elevated expression for nLc4 (25–98%). A distinct expression of nLc4 (5–43%) was observed in non–ovarian derived cancer cells (Fig. 1B). P_1_ expression was observed only in IGROV1 (27%) and Ls174T (23%) cell lines (Fig. 1B). Based on their nsGSL expression levels, IGROV1 was selected for genome editing to establish a heritable and site-specific *B3GNT5* knockout cell line (∆*B3GNT5*), which was then selected to study the influence of nsGSLs on the glycome repertoire.

### Genome editing of *B3GNT5* for depletion of nsGSLs and validation using flow cytometry

**Figure 2:**
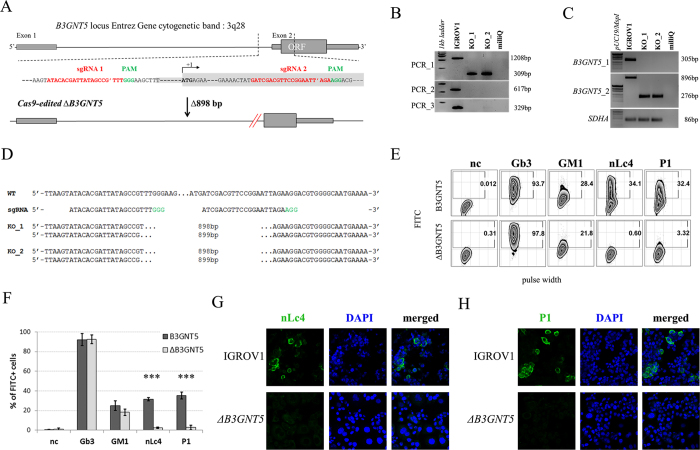
Deletion of *B3GNT5* leads to depletion of nLc4 and P_1_. (**A**) *B3GNT5*-editing strategy using two different sgRNAs (red) contain PAM sequence (green) deleting a genomic region up- and downstream of the transcription start site (+1) including a part of the open reading frame (ORF). *In silico* analysis revealed a deletion of 898 bp. (**B**) Representative genotyping PCR for characterization of single cell clones. PCR numbers indicate the use of different primer pairs listed in experimental procedures. (**C**) Semi-quantitative PCR with two different primer pairs (*B3GNT5*_1 and *B3GNT5*_2) on wildtype (pIGROV1) and two representative knockout clones. *SDHA* was used as a reference gene. (**D**) DNA sequencing results for wildtype (WT), and selected biallelically deleted knockout clones (KO_1 and KO_2). (**E**) Representative counter plots for presence of Gb3, GM1, nLc4 and P_1_ before (wildtype *B3GNT5*) and after gene disruption of *B3GNT5* in IGROV1 cells (∆*B3GNT5*). Percentage of GSL-positive cells (FITC) is shown in each plot. Negative control (n.c.). (**F**) Box and Whisker plots representing CRISPR *Cas9*-mediated changes in expression of individual GSLs (percentage of FITC-positive cells, ordinate) for ∆*B3GNT5* and parental cells (abscissa). ***p < 0.001 was obtained by two-way ANOVA. Data are representative out of four independent experiments. (**G**,**H**) Confocal fluorescence images displaying presence and absence of nLc4 (green, G, 40x magnification) and P_1_ (green, H) in *B3GNT5* wildtype (*B3GNT5*) and knockout (∆*B3GNT5*) cells, respectively. Cells are counterstained with DAPI (blue). Data are represented as mean ± s.d.

*B3GNT5* is the key glycosyltransferase involved in synthesis of nsGSLs^6^,^18^. We utilized the genome editing technology, CRISPR-*Cas9*, to homozygously delete an 898 bp genomic region, including the translation start site of *B3GNT5* (Fig. 2A). The plasmid pSpCas9(BB)-2A-GFP encoding two specific sgRNA sequences (Supplemental Table S1), was transiently transfected into IGROV1 and tested after 72 h incubation for *Cas9* activity, in which an additional band at 309 bp (∆*B3GNT5*) observed below 1208 bp (wildtype *B3GNT5*) indicates an active genome editing. Next, *Cas9*-active cell pools were subjected to single cell sorting (Supplemental Fig. S1) and incubated until further genotyping of single cell clones. The presence of homozygous ∆*B3GNT5* was verified by three independent PCRs, which showed the additional band at 309 bp in knockout (PCR_1) and no visible band at 617 bp and 329 bp, respectively for wildtype *B3GNT5* (PCR_2 and PCR_3) (Fig. 2B). We identified two homozygous *B3GNT5*-deleted clones from a total of 320 clones profiled (0.625% efficiency). Despite equal mRNA expression of *B3GNT5* transcripts (B3GNT5_1), a truncated transcript length was equally expressed in ∆*B3GNT5* cells as compared to wildtype (B3GNT5_2, Fig. 2C). The homozygous deletion was confirmed by Sanger DNA sequencing, showing knockout cells with alleles in varying lengths; (*indel*) 898 bp and 899 bp (Fig. 2D). In regards to potential off-target effects, both sgRNAs used for genome- editing did not show off-target effects (Supplemental Fig. S2).

The established ∆*B3GNT5* cell line was further investigated for GSL expression using flow cytometry, in which the human anti-P_1_ IgM P3NIL100 antibody, previously validated by printed glycan array, was utilized to detect the binding specificities to P_1_ epitope on these cells (Supplemental Fig. S3)^14^. The GSL pathways affected by the genetic disruption of *B3GNT5* is hypothesized (according to the scheme presented in Fig. 1A) and the binding results were in full concordance with the expression levels for nLc4 and P_1_ (*p *<* *0.001, Fig. 2E and F). In conclusion, the absence of nLc4 and P_1_ epitopes confirmed our hypothesis that the nsGSLs were depleted in these cells, whereas ganglio- and globo- series GSLs were not affected by *B3GNT5*-editing (Fig. 2E and F). In addition, the absence of nLc4 and P_1_ in ∆*B3GNT5* cells was confirmed by confocal fluorescence microscopy (Fig. 2G and H).

### Validation of altered GSL-glycans in *∆B3GNT5* cells by PGC-UHPLC-ESI-QTOF-MS/MS

We also utilized mass spectrometry (MS) to investigate the expression of cell surface glycans, which were subsequently altered due to the genome- editing of *B3GNT5* in IGROV1 cells. This glycomics-based approach was performed to confirm the absence of nsGSLs due to genetic disruption of *B3GNT5*. Glycans were enzymatically released from extracted GSLs of parental and ∆*B3GNT5* IGROV1 cells and analyzed using negative mode UHPLC-ESI-QTOF-MS/MS. The assignment of glycan structures was facilitated using diagnostic MS^2^ fragment ions previously described for the analysis of *N*-glycans released from glycoproteins as well as GSL-derived glycans using negative mode LC-ESI-MS/MS^13^,^19^.

**Figure 3:**
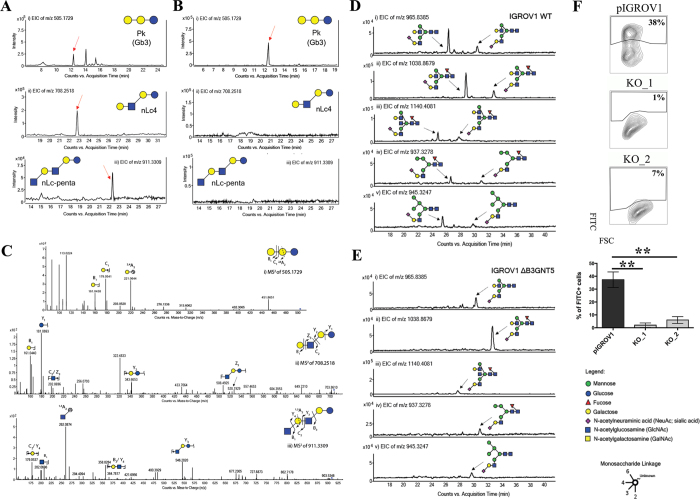
LC-MS profiling of neutral GSLs and monosialylated *N*-glycans extracted from wildtype IGROV1 and genome-edited B3GNT5 cell lines. The extracted ion chromatograms (EIC) obtained from wildtype IGROV1 (**A**) and genome-edited B3GNT5 (**B**) cell lines are represented for Gb3 [*m*/*z* 505.1729^1−^Galα1-4Galβ1-4Glcβ1] (i), nLc4 [*m*/*z* 708.2518^1−^:Galβ1-4GlcNAcβ1-3Galβ1-4Glcβ1] (ii) and nLc-pentasaccharide [*m*/*z* 911.3309^1−^:GlcNAcβ1-3-Galβ1-4GlcNAcβ1-3Galβ1-4Glcβ1] (iii). (**C**) MS^2^ spectrum of the precursor ion at *m*/*z* 505.1729^1−^, *m*/*z* 708.2518^1−^ and *m*/*z* 911.3309^1−^ derived from Gb3 (i), nLc4 (ii) and nLc-penta (iii), respectively. PGC- LC allows for the separation of α2-6 and α2-3 sialylated *N*-glycans based on retention time. (**D**) The EICs obtained from the wildtype IGROV 1 depict three major monosialylated complex *N*-glycans at *m*/*z* 965.8385^2−^ [(Neu5Ac)_1_(Gal)_2_(GlcNAc)_2+_ (Man)_3_(GlcNAc)_2_] (i), *m*/*z* 1038.8679^2−^ [(Neu5Ac)_1_ (Gal)_2_(GlcNAc)_2_(Fuc)_1+_ (Man)_3_(GlcNAc)_2_] (ii) and *m*/*z* 1140.4081^2−^ [(Neu5Ac)_1_(Gal)_2_ (GlcNAc)_3_(Fuc)_1_ + (Man)_3_(GlcNAc)_2_] (iii) and two monosialylated hybrid *N*-glycans at *m*/*z* 937.3278^2−^ [(Neu5Ac)_1_(Gal)_1_(GlcNAc)_1_(Man)_1_ + (Man)_3_(GlcNAc)_2_] (iv) and *m*/*z* 945.3247^2−^ [(Neu5Ac)_1_(Gal)_1_(GlcNAc)_1_(Man)_2_ + (Man)_3_(GlcNAc)_2_] (v) which display α2–3 and α2–6 sialylated isomers at separate retention times. (**E**) The EICs of genome-edited B3GNT5 cells depicts the loss of the α2–6 sialylated isomer for all five of the above mentioned monosialylated complex [*m*/*z* 965.8385^2−^ (i), *m*/*z* 1038.8679^2−^(ii), *m*/*z* 1140.4081^2−^ (iii)] and hybrid [*m*/*z* 937.3278^2−^ (iv), *m*/*z* 945.3247^2−^ (v)] *N*-glycans. (**F**) SNA staining confirmed reduction of α2–6 sialylation in ∆*B3GNT5* cells. Representative counter plot and barograph summarizing three independent experiments on parental IGROV1 cells (pIGROV1) and ∆*B3GNT5* cells (KO_1 and KO_2); **p-value < 0.01. Data are represented as mean ± s.d.

The MS profiling revealed three neutral GSLs comprising of globo (Gb3)-, paragloboside (nLc4) as well as neolactopentaosylceramide (nLc-penta). All three neutral GSL species differed in chromatographic retention time, as well as MS^2^ fragmentation patterns facilitating the structural assignment of the GSL-glycans based on the fragment ions arising from various glycosidic and cross-ring cleavages. Table 1 shows the list of GSL-glycans and their relative intensities that were observed in both cell lines. The globo series glycosphingolipid, Gb3, was detected as [M-H]^1−^ *m*/*z* 505.1729^1−^ in wildtype and ∆*B3GNT5* cells and was shown to elute at 12.16 min (Fig. 3A(i) and B(i)). The representative MS^2^ spectra of the precursor ion at *m*/*z* 505.1729^1−^ (Fig. 3C(i)) showed prominent glycosidic- type fragment ions (B_1_ at *m*/*z* 161.0438^1−^, C_1_ at *m*/*z* 179.0541^1−^) corresponding to the Gal-Gal-Glc trisaccharide sequence. The presence of the 4-linked terminal Gal to the inner (Gal-Glc) disaccharide was further confirmed by the characteristic cross ring fragment ion corresponding to ^2,4^A_2_ at *m*/*z* 221.0644^1−^. The second glycan structure, paragloboside (nLc4), detected as [M-H]^1−^ *m*/*z* 708.2518^1−^ was found to be present only in the wildtype IGROV1 cell line but not in the ∆*B3GNT5* cell line. This glycan was shown to elute at 22.60 mins (Fig. 3A(ii)) and the MS^2^ spectra comprised of a mixture of B and Y ions (B_1_ at *m*/*z* 161.0440^1−^, Y_1_ at *m*/*z* 181.0693^1−^, Y_2_ at *m*/*z* 343.6653^1−^) as well as C and Z ions (Z_3_ at *m*/*z* 528.1929^1−^, C_2_/Z_3_ at *m*/*z* 202.0696^1−^) that corresponded to the tetrasaccharide sequence, Hex-HexNAc-Gal-Glc (Fig. 3C(ii)). The absence of the cross ring cleavage ions characteristic of 4-linked GalNAc corresponding to ^0,2^A_3_ and ^2,4^A_3_ at *m*/*z* 484.1672^1−^ and *m*/*z* 424.1460^1−^, respectively, further confirmed the presence of the 3-linked GlcNAc residue to the inner Gal-Glc of the tetrasaccharide. The late elution time observed for this glycan structure has been reported in our previous study demonstrating a similar retention time for nLc4 identified from IGROV1 cell lines using PGC-LC-ESI-IT-MS/MS (20). We also identified a third glycan structure at [M-H]^1−^ *m*/*z* 911.3309^1−^ in wildtype IGROV1 which was shown to elute at 22.10 min (Fig. 3A(iii) and B(iii)). The MS^2^ spectra comprised of mainly B-, C- and Y-type fragment ions (B_1_ at *m*/*z* 202.0696^1−^, C_2/_Y_4_ at *m*/*z* 179.0537^1−^, B_3/_Y_4_ at *m*/*z* 364.7817^1−^ and Y_3_ at *m*/*z* 546.2020^1−^) that corresponded to the tentative pentasaccharide sequence, HexNAc-Gal-HexNAc-Gal-Glc corresponding to a poly-LacNAc-type pentaosylceramide (Fig. 3C(iii)). The presence of the ^1,3^A_2_ at *m*/*z* 262.0874^1−^ further confirmed the terminal GlcNAc linked to the internal tetrasaccharide. However, this structure was not observed in the MS spectra of the ∆*B3GNT5* cell line. Whilst we were able to confirm the absence of nLc4 in the ∆*B3GNT5* cell line, the presence of the P_1_ epitope was not readily detected in both the IGROV1 and ∆*B3GNT5* cells analyzed in this study. This could be due to the low expression of the P_1_ epitope, coupled with the use of different MS instrumentation in this study (as compared to our previous study using PGC-LC-ESI-IT-MS/MS that was performed on P_1_ – enriched cell populations of IGROV1^13^). Moreover, this study used a larger PGC column inner diameter on a conventional LC platform performed which resulted in less sensitivity as opposed to the microflow LC platform performed previously using PGC-ESI-IT-MS/MS. Apart from the neutral GSLs, two gangliosides were also detected in the MS spectra of both the wildtype and ∆*B3GNT5* cells. Both GM3 and GM2 were detected as [M-H]^1−^ *m*/*z* 634.2142^1−^ and [M-H]^1−^ *m*/*z* 837.2926^1−^, respectively, while the third ganglioside, GM1, at [M-H]^1−^ *m*/*z* 999.3548^1−^, did not appear to be present in both the cell lines (Table 1). These results indicate that gangliosides remained unaffected by the genome-editing of *B3GNT5*.

### Isomeric *N*-glycan profiling reveals concomitant loss of α 2-6 sialylation on membrane proteins in B3GNT5-mediated depletion of nsGSL

In addition to the profiling of the GSLs, we also compared the *N*-glycan profiles of the wildtype and ∆*B3GNT5* cell lines to gain an insight into the glycan structural changes, which could potentially be affected as a result of the GSL-related *B3GNT5* genome- editing. We have previously reported an increased relative abundance of α2-6 sialylation as compared to α2-3 sialylation on membrane *N*-glycans of IGROV1^19^. The presence of these isomeric structures in *N*-glycans can be differentiated based on their retention time on the porous graphitized carbon (PGC)-LC column, whereby glycans bearing the α2-6 sialic acid isomer are less strongly retained on the column and thus elute much earlier as compared to the α2-3 sialylated *N*-glycans. Likewise, in this study, a similar glycan elution pattern was observed in the wildtype IGROV1 cell lines confirming our previous results (which used another mass spectrometry setup), in which all five *N*-glycans comprising of the sialylated complex (*m*/*z* 965.8385^2−^, *m*/*z* 1038.8679^2−^, *m*/*z* 1140.4081^2−^) (Fig. 3D(i–iii)) and hybrid (*m*/*z* 937.3278^2−^ and *m*/*z* 945.3247^2−^) (Fig. 3D(iv–v)) glycans were found to contain α2-6 and α2-3 isomeric structures which eluted at separate retention times. Surprisingly, we noted that in comparison to the wildtype IGROV1, the genome- edited ∆*B3GNT5* cells were shown to display only the α2-3 sialylated glycans but not the α2-6 sialylated *N*-glycan isomer for all five of the *N*-glycan masses described above (Fig. 3E(i–v)). We also profiled the membrane protein *O*-glycans of both cell lines to see if the difference in the sialylation pattern could be attributed to a global glycosylation change across all glycan types. However, we observed no change in the *O*-glycan sialylation profiles, further demonstrating that the preferential α2-6 sialylation was specific to only the *N*-glycosylation pathway, as a result of the *B3GNT5* genome- editing. The *N*- and *O*-glycans identified in both cell lines and their corresponding relative intensities are shown in Table 2.

In order to confirm the reduction of α2-6 sialylation on membrane glycoproteins, we performed a staining using *Sambucus nigra* agglutinin lectin (SNA), which has been shown to bind preferentially to sialic acid attached to terminal galactose in *via* α2-6 linkage^20^. The parental IGROV1 revealed positive staining (37% ± 6% FITC^+^) for α2-6Neu5Ac whereas for both ∆*B3GNT5* cells, the expression of α2-6 –linked Neu5Ac was significantly reduced (KO_1 = 2% ± 1.7% and KO_2 = 6% ± 2.6% FITC^+^) (*p *<* *0.01; Fig. 3F), thereby confirming the loss of membrane protein α2-6 sialylation.

### α2-6 sialylation is not affected in B4GALNT1 ganglioside-depleted IGROV1 cells

**Figure 4:**
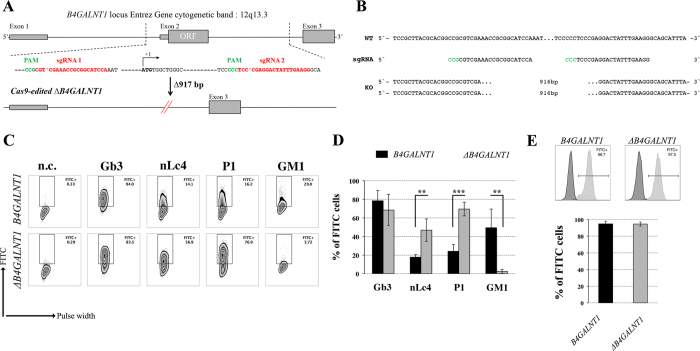
Loss of gangliosides by *B4GALNT1*-editing does not affect α2–6 sialylation in IGROV1 cells. (**A**) Depiction of CRISPR-*Cas9*-mediated *B4GALNT1* editing in IGROV1 cells using two different sgRNAs [red; PAM sequence (green)]. *In silico* analysis revealed a deletion of 917 bp including translation start site located at exon 2. (**B**) Verification of ∆*B4GALNT1* cells by DNA sequencing. (**C**) Representative flow cytometry zebra blot for parental IGROV1 and ∆*B4GALNT1* cells. (**D**) Bar chart summarizing three independent flow cytometry experiments. (**E**) SNA-staining remains unaffected in ∆*B4GALNT1* cells compared to IGROV1 (*B4GALNT1*). Data are represented as mean ± s.d.

Our current data demonstrate that the deletion of *B3GNT5* and consequently, the loss of nsGSLs, have unexpectedly led to the observation that α2-6 sialylation is reduced on *N*-glycosylated proteins. To investigate whether this effect can be also observed when other GSL series are genome-edited, we utilized the CRISPR-*Cas9* to homozygously delete the *B4GALNT1* gene encoding for the beta-1,4-*N*-acetyl-galactosaminyltransferase-1 glycosyltransferase involved in the synthesis of gangliosides. IGROV1 cells were transfected with two sgRNA *Cas9*-encoding constructs to delete exon 2 of *B4GALNT1*, resulting in a 917 bp deletion (Fig. 4A). Clones identified as homozygously edited *B4GALNT1* knockout cells (*ΔB4GALNT1*) were confirmed by DNA sequencing based on the deletion of 916 bp (Fig. 4B). Following measurement of GSL expression, as expected, we observed a significant reduction for GM1 (*p *<* *0.01) and no change in the expression of globoside Gb3 (Fig. 4C and D). In line with a previous report showing appearance of nLc4 after sialidase treatment in ganglioside-depleted mice^21^, we found that nLc4 and P_1_ are significantly elevated in Δ*B4GALNT1* cells (*p *<* *0.01, Fig. 4D). In regards to α2-6 sialylation, we did not observe a difference in SNA staining to IGROV1 cells compared to Δ*B4GALNT1* cells (*p *>* *0.05, Fig. 4E). Taken together, CRISPR-*Cas9* mediated disruption of *B4GALNT1* leading to loss of gangliosides did not subsequently alter α2-6 sialylation on glycosylated proteins in contrast to that observed for Δ*B3GNT5* cells.

### Loss of α2-6 sialylation is a result of silenced *ST6GAL1* expression in *∆B3GNT5* cells

**Figure 5:**
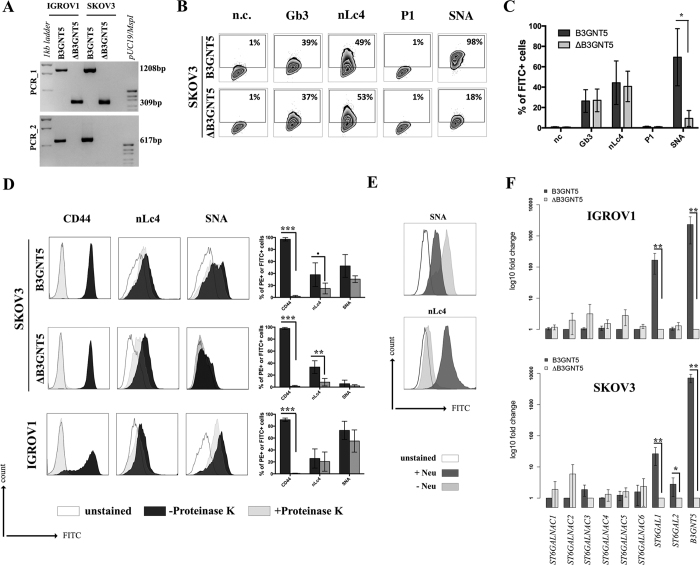
The reduction of α2-6 sialylation occurs in two *∆B3GNT5* ovarian cancer cell lines. (**A**) Representative genotyping PCR for ∆*B3GNT5* SKOV3 ovarian cancer cells. PCR_1, wildtype-specific PCR; PCR_2, deletion specific PCR. (**B**) Representative zebra plot for GSL expression in SKOV3 wildtype and ∆*B3GNT5* cells. (**C**) Quantified data from three independent flow cytometry experiments. (**D**) The epitope for anti-nLc4 antibodies is present on glycoproteins and glycosphingolipids in SKOV3 and IGROV1 cells, respectively. Proteinase K treatment on SKOV3, SKOV3 ∆*B3GNT5* and IGROV1 cells following staining for CD44, nLc4 and α2-6 neuraminic acid (with SNA). Representative histogram for unstained (white), proteinase K untreated (dark gray) and proteinase K treated cells (light gray). Bar chart summarizes three independent experiments. ***p < 0.001, **p < 0.01, *p-value < 0.05,*p*-value < 0.1. (**E**) Histogram for IGROV1 cells treated with neuraminidase and stained for α2-6 neuraminic acid (with SNA) and nLc4; unstained (white), neuraminidase treated (dark gray) and untreated cells (light gray). (**F**) Relative expression of genes encoding glycosyltransferase known to attach α2-6 neuraminic acid to glycoproteins and glycosphingolipids. Bar chart shows the mean and standard deviation of three independent experiments of target genes (*ST6GALNAC1-6, ST6GAL1, ST6GAL2*, and *B3GNT5*) in wildtype (IGROV1 and SKOV3) and corresponding ∆*B3GNT5* cells. Data are represented as mean ± s.d.

The reduction of α2-6 sialylation in ∆*B3GNT5* IGROV1 cells, prompted us to investigate whether this observation is constrained to a specific cell line or reproducible in another ovarian cancer cell line. Thus, we applied the genome-editing strategy as reported for IGROV1 (Figs 2A and 5A) in SKOV3 cells, which were chosen based on their expression of nLc4 and α2-6 sialic acid (Figs 1B and 5B). In contrast, Ls174T being positive for nLc4 and P_1_ did not show α2-6 sialylation and was therefore not selected for genome- editing (Supplemental Fig. S4A). Analysis of the GSL expression in parental SKOV3 and corresponding ∆*B3GNT5* cells revealed only marginal changes for Gb3 and nLc4, whereas SNA staining was significantly reduced (*p* = 0.0232). This confirms what was previously observed in ∆*B3GNT5* IGROV1 cells, and further indicates that the disruption of *B3GNT5* leads to a reduction in α2-6 sialylation and may be a cell line-independent phenomenon (Fig. 5B and C).

It is interesting to note, however, that we did not observe any reduction of nLc4 in SKOV3 ∆*B3GNT5* cells (Fig. 5C). Thus, we hypothesized that besides GSLs, the antibody may also recognize the terminal nLc epitope (Galβ1-4GlcNAc) on *N*- and *O*-glycoproteins carrying Type II LacNAc (Galβ1-4GlcNAc) terminated antennas and therefore, the binding levels in SKOV3 Δ*B3GNT5* cells remained unchanged. To analyze whether the antibody-binding epitope is also carried on cell surface proteins, we treated SKOV3, SKOV3 Δ*B3GNT5*, and IGROV1 cells with Proteinase K and performed staining for nLc4, SNA, and CD44. The latter was used as a control antigen (sensitive to Proteinase K treatment). As shown in Fig. 5D, cells subjected to Proteinase K treatment showed a complete loss of CD44 epitope, an ubiquitously expressed membrane protein, indicating that proteins on the cell surface are fully digested. In contrast, SKOV3 and SKOV3 Δ*B3GNT5* cells showed reduction of nLc4 staining after Proteinase K treatment whereas IGROV1 cells remained positive after nLc4 staining. This suggests that the nLc4 epitope was potentially present on cell surface proteins on SKOV3 as well as Ls174T cells (Supplemental Fig. S4B). In regards to α2-6 sialylation, Proteinase K treated cells showed only marginal reduction in SNA staining (Fig. 5D). Finally, IGROV1 cells (no reduction upon Proteinase K treatment for nLc4) were also treated with a broad-specificity neuraminidase to remove α2-6/2-3-linked Neu5Ac, resulting in the reduction of SNA staining (Fig. 5E). This observation corroborated with a further increase in nLc4 staining, indicating that nLc4 on GSLs is terminated with Neu5Ac to form sialyl- 3 or 6- paragloboside GSLs in IGROV1 cells and is further exposed on the cell surface upon de-sialylation. Taken together, our results provide evidence that apart from nLc4 expression on GSLs, the anti-nLc4 antibody epitope is also preferentially expressed on glycoproteins in SKOV3 cells, as indicated by the sustained presence of nLc4 in SKOV3 Δ*B3GNT5* cells and terminal nLc4 in GSLs may further be modified to form sialo-paraglobosides, as observed in IGROV1 cells.

**Figure 6:**
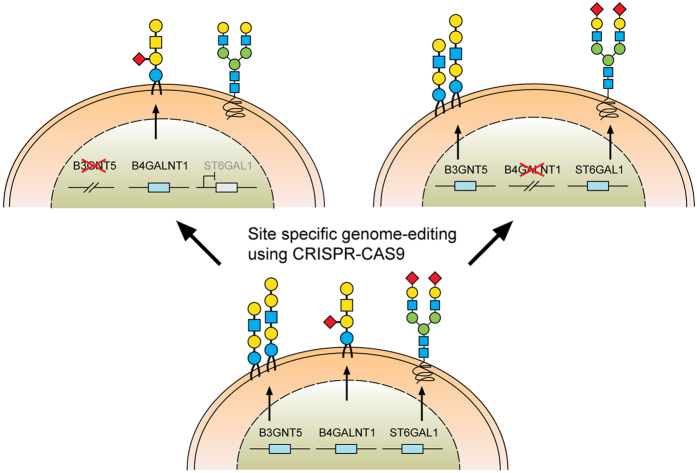
A model summarizing the interaction of specific glycolipid synthases on glycosphingolipids and *N*-glycosylation of proteins.

Next, we investigated whether mRNA expression of genes encoding sialyltransferases involved in the synthesis of α2-6-sialoglycans is altered upon deletion of *B3GNT5*. RT-qPCR was established in concordance with MIQE guidelines^22^ and glyco-gene expression was normalized to the geometric mean of three independent reference genes (Supplemental Table S2). We profiled *ST6GALNAC1-6* and *ST6GAL1-2* in parental IGROV1 and SKOV3 as well as their corresponding ∆*B3GNT5* cells. Apart from the expected change in *B3GNT5* mRNA levels, *ST6GAL1* expression was found to be significantly decreased in both ∆*B3GNT5* cells of up to 166-fold for IGROV1 (Fig. 5F). The expression of the remaining sialyltransferases encoding genes examined was not significantly altered in the wildtype and ∆*B3GNT5* cells, except for *ST6GAL2* in SKOV3 cells (Fig. 5F). In addition, *ST6GAL1* expression in parental and IGROV1 *∆B4GALNT1* cells revealed equal gene expression levels (Supplemental Fig. S5), corroborating with no changes observed in SNA staining. In conclusion, these results indicate that the disruption of *B3GNT5* and the corresponding loss of α2-6 sialylation on *N*-glycans appear due to the preferential silencing of *ST6GAL1* gene expression in both ovarian cancer cell lines, IGROV1 and SKOV3 (Fig. 6).

## Discussion

The advent of novel nuclease-based precision genome- editing techniques is starting to revolutionize the field of glycobiology, enabling stable gene editing and potentially unravelling the roles of glyco-related genes and their corresponding glycosyltransferases in mammalian cell lines. This technology was first demonstrated by Henrik Clausen and his colleagues, through the introduction of the ‘Simple Cell’ strategy, to study the role of truncated *O*-glycans in malignant diseases^23^,^24^, specificity of anti-glycopeptide antibodies^25^, profile and map the human *O*-GlcNAc glycoproteome^26^,^27^, and produce novel antibodies to defined glycopeptides^28^. Whilst a majority of this work is based on *O*-glycans, there is very limited information on the use of genome- editing for glycosphingolipids (GSLs) and in particular, the nsGSLs. Here, we utilized CRISPR-*Cas9* nuclease genome- editing to explore the role of *B3GNT5*, the key glycosyltransferase responsible for the synthesis of precursor Lc3 that is extended to form the lacto (Type 1) and neo-lacto (Type II) series GSLs^6^. For the first time, using a combination of genomic-level editing and cell surface membrane protein and GSL-glycan profiling, we provide valuable insights into the complex interplay regulating the expression of linkage-specific α2-6 sialo-*N*-glycans on proteins as a consequence of GSL disruption. We demonstrate that by mutating *B3GNT5*, the depletion of nsGSLs consequently affects α2-6 sialylation on *N*-glycoproteins, not only at the cellular level, but also through the silencing of the corresponding *ST6GAL1* gene.

As evidenced in this study, the genomic deletion of *B3GNT5* resulted in the loss of the (neo-) lacto paragloboside (nLc4, Galβ1-4GlcNAcβ1-3Galβ1-4Glcβ1-1Ceramide) as well as P_1_ (Galα1-4Galβ1-4GlcNAcβ1-3Galβ1-4Glcβ1-1Ceramide), a pentasaccharide moiety of which has not been annotated in the KEGG database so far. We recently reported the presence of the P_1_ glycan in tissue samples^14^ as well as on P_1_-enriched IGROV1 ovarian cancer cell lines^13^, serving as a cell-recognition molecule through its binding to naturally occurring anti-glycan antibodies^14^,^29^. The presence of nLc4 and P_1_ was detected on IGROV1 cells using flow cytometry, in which P_1_ was detected using P3NIL100 antibody, a well-validated monoclonal IgM antibody shown to specifically recognize the chemically synthesized P_1_ trisaccharide (Galα1-4Galβ1-4GlcNAcβ-), as indicated by glycan array profiling performed in this study. Both the flow cytometry and high-resolution ESI-QTOF-MS and MS/MS profiles confirmed that the levels of nLc4 were reduced, while the globo (Gb3) and ganglio (GM3, GM2 and GM1)- series GSL glycans remained unaffected in ∆*B3GNT5* cells. The presence of P_1_, however, was not detected on the parental IGROV1 cells by the mass spectrometry approach applied, possibly due to the low detection levels of P_1_ on non-P_1_ enriched IGROV1 cells used in this study, as well as the use of a different mass spectrometric platform. Nevertheless, we also observed that apart from IGROV1, the colon cancer cell line, Ls174T, showed detectable levels of P_1_ providing further evidence that P_1_ is not exclusively present on erythrocytes^30^,^31^,^32^. The results obtained herein support that the pentasaccharide P_1_ is indeed exclusively present as an nsGSL-glycan on membrane glycolipids and not on glycoproteins.

To date, it remains unclear if nLc4- and P_1_- GSL glycans are functionally relevant molecules in diseased states such as cancer and infection or if they are ubiquitously expressed. In regards to pathogenic infections, it has been previously shown that *Helicobacter pylori* induces *B3GNT5* expression and that the ABO(H)/Lewis blood group antigens expressed in *H. pylori*- infected individuals act as receptors for BabA, thereby facilitating colonization of the gastric niche^33^. Likewise, the *Shiga* toxin from urinary tract infection-causing enterohemorrhagic *E. coli* was shown to recognize the terminal epitope of P_1_, Galα1-4Galβ1-4 following internalization of the toxin by receptor-mediated endocytosis^34^. Another study also showed that the *Shiga*-like toxin binds to P_1_ trisaccharide on core 2 *O*-glycoproteins in overexpressed CHO cells^35^. These findings indicate that this toxin may recognize P_1_ pentasaccharide. It is evident that the successful *B3GNT5* deletion performed in this study may be further applied to investigate the lectin binding specificities (*e.g. Shiga* toxin) in other human cancer cell lines expressing Gb3, nLc4 and P_1_.

Perhaps, the most intriguing finding from this study is the unexpected loss of α2-6 sialylation observed using mass spectrometry in ∆*B3GNT5* cells which was further substantiated by SNA lectin staining on cultured cells and reduced *ST6GAL1* mRNA expression. We have previously shown in two separate studies, respectively, that ovarian cancer cells, IGROV1 and SKOV3, have higher levels of glycoprotein α2-6 sialylation as compared to non-cancer ovarian epithelial cells^19^ and nLc4 and P_1_ are both expressed on IGROV1 and ovarian cancer tissue-derived GSL-glycans^13^. Hence, the reduction of α2-6 sialylation as a direct consequence of the *B3GNT5* deletion in ovarian cancer cells is indicative of their complex associations within the glycan-processing pathway. A recent publication reported a similar finding in the context of the rare autosomal recessive salt and pepper syndrome^36^. In this study, a homozygous transition mutation in the gene encoding sialyltransferase ST3GAL5 (GM3 synthase) was described. More importantly, in addition to the reduced GSL complexity, the comprehensive glycomics analysis performed using mass spectrometry also revealed altered *N*- and *O*-glycans on proteins due to the point mutation in the *ST3GAL5*^36^. In another study on respiratory chain disorders, the authors observed significant increases of Gb3 and Gb4 and a decrease of LacCer in fibroblasts^36^,^37^. Similarly, the appearance of unexpected gangliosides in homozygously deleted *ST3GAL5* murine primary embryonic fibroblast cells was also reported^38^. Despite the limited number of publications, our current study provides another example on how a loss of a specific glycosyltransferase interferes with glycomic changes, as observed in the reduction of α2-6 sialylation on *N*-glycoproteins, thereby recognizing the contribution of altered GSL expression in cell development and function. It is not known whether the loss of α2-6 sialylation is thought to provide a compensation phenotype for the depleted nsGSLs in these cells or enables them to survive despite the glycan changes. It is also unclear as to how the ST6GAL1 is regulated in the context of their translocation within the Golgi membrane or their accessibility to the membrane proteins. It will be useful to further characterize membrane proteins bearing these sialylated *N*-glycans and observe corresponding changes in the proteome profiles of *B3GNT5* –edited ovarian cancer cells.

Of note, despite similar observations in two independent ovarian cancer cell lines and due to our carefully designed sgRNAs in a way to minimize the probability of off-target activity; we cannot exclude unintended changes in the genome of our genome- edited ovarian cancer cell lines. Here, an unbiased approach would be necessary to exclude mutations anywhere in the genome^39^. Therefore, the most reliable method would be whole genome sequencing^40^. A direct *in situ* breaks labeling, enrichment on streptavidin, and next-generation sequencing also referring to BLESS^41^ as well as linear amplification-mediated high-throughput genome-wide translocation sequencing (HTGTS)^42^ can also be applied as less expensive alternatives to track genomic alterations caused by off target *Cas9*-activity.

In conclusion, the investigation of CRISPR-*Cas9*-mediated deletion of glycosyltransferase in ovarian cancer cells, coupled with the use of MS-based glycomics profiling, serve as a useful model for future studies to elucidate the biological roles of nsGSLs, an important group of GSLs which may be involved in specific cell physiology functions. More importantly, the prominent changes in *N*-glycoprotein sialylation detected in the *B3GNT5* genome- edited cells are reflective of global alterations in one or more regulatory components essential for glycan biosynthesis, thereby warranting further research efforts into the complex and overlapping roles of glycosyltransferases involved in the downstream glycan biosynthesis pathways.

## Methods

### Cell culture

Cell lines (HOSE17.1, IGROV1, SKOV3, BG1, CAOV3, Ls174T, HeLa, HCT15, and HCT116) were grown in RPMI1640 media supplemented with 10% fetal bovine serum (FBS), penicillin (100 U/ml) and streptomycin (100 μg/ml) (Sigma-Aldrich, Buchs, Switzerland). Fallopian tube cell lines FT33 Tag, FT190, and FT237 (kind gifts by Dr. Drapkin) were cultured in DMEM F12/50 without HEPES supplemented with 2% (v/v) Ultroser (USG, Pall Corporation, USA) and penicillin/streptomycin. All cell lines were cultured at 37 °C in a 95% humidified atmosphere containing 5% CO_2_. Cell lines were short tandem repeat (STR) profiled and routinely tested for mycoplasma infection^43^.

### Antibodies and reagents

Primary antibodies to detect the cell surface-associated GSLs were applied as described previously^15^. Briefly, the following primary antibodies were utilized; rat IgM anti-P^k^ (Gb3) antibody (ABDserotec, Germany); mouse IgM anti-nLc4 (paragloboside) antibody [clone 1B2 kindly provided by Prof. Mandel and Prof. Clausen, Denmark]^44^; human IgM anti-P_1_ antibody (Millipore, Germany), and Cholera toxin B subunit (Sigma-Aldrich, Switzerland) to detect GM1. All secondary antibodies (mouse anti-rat IgM antibody conjugated to biotin, rat anti-mouse IgM conjugated to biotin and mouse anti-human IgM conjugated to biotin) were obtained from BD Pharmingen. Cell surface GSL expression was visualized with FITC conjugated to streptavidin (BD Pharmingen). Non-viable cells were positively stained with 7-amino-actinomycin D (BD Pharmingen). Isotype controls for each primary antibody used included the following: purified rat IgM (BD Pharmingen), purified mouse IgM and chrompure human IgM (Jackson ImmunoResearch, USA). Alpha 2-6 sialylation on the cell surface was investigated using *Sambucus nigra* lectin (SNA) conjugated to biotin (Vector Laboratories, Reactolab, Servion, Switzerland). Lectin staining was visualized with streptavidin conjugated to FITC. Anti human CD44-PE antibody was purchased from Miltenyi Biotec (Bergisch Gladbach, Germany) for use as a positive control in Proteinase K treatment.

### CRISPR-Cas9 sgRNA design and construction

The design of sgRNAs was carried out using the online program available from Zhang’s laboratory^45^. This program provides additional information about the quality of sgRNA by giving it a score value of up to 100 and additionally, the number and sites of potential off targets. Designed and processed sgRNAs to edit either *B3GNT5* or *B4GALNT1* (Supplemental Table S1) were cloned into pSpCas9(BB)-2A-GFP (addgene PX458) *via BsbI* restriction site using T4 DNA Ligase (Promega, Dübendorf, Switzerland).

IGROV1 and SKOV3 cells were transfected using ViaFect Transfection reagent (Promega, Dübendorf, Switzerland). A total of 4–5 × 10^5^ cells were seeded into 6-well plates, cultured for 24 h and transfected with 2.5 μg of pSpCas9(BB)-2A-GFP. Cells were harvested after 72 h and subjected to single cell sorting.

### Flow cytometry-based single-cell sorting

IGROV1 transfected with pSpCas9(BB)-2A-GFP targeting either *B3GNT5* or *B4GALNT1* were grown for 72 h, washed twice in PBS and harvested using non-enzymatic cell dissociation buffer (Sigma-Aldrich, Buchs, Switzerland). Cells were then resuspended in RPMI containing 10% FCS before single cell sorting was performed on a BD FACS Vantage SE DiVa Cell Sorter (BD Biosciences). Cells sorted for single DAPI^−^ and GFP^+^ cells were seeded into 96-well flat-bottom plates with pre-warmed RPMI containing 10% FCS. Plates were incubated for 2 to 3 weeks following transfer to 48-well plates, prior to genomic DNA isolation for genotyping PCR to characterize single cell clones.

### Genotyping PCR

Selected clones were characterized by three to four PCRs using appropriate primer pairs (Supplemental Table S1). PCR was performed using 1x GoTaq Green Master Mix (Promega, Dübendorf, Switzerland), 200 nM primer (Supplemental Table S1) and 30 ng gDNA and carried out on a Biometra Professional Trio cycler. PCR was performed under the following conditions: initial denaturation at 94 °C for 1 min followed by 33 cycles consisting of denaturation at 94 °C for 15 sec, annealing at 59 °C for 15 sec, and elongation at 72 °C for 15–45 sec depending on the amplicon length. The PCR products were separated on a 1.7% agarose gel.

### Sanger DNA Sequencing

PCR products were cloned into pGEM^®^-T Easy Vector System (Promega) according to the manufacturer’s protocol. DNA sequencing was performed by Source Bioscience Life Sciences (Berlin, Germany). Samples were shipped at a concentration of 100 ng/μl with specific primer (if required) concentration of 3.2 pg/μl.

### Off target screening

Potential off target sites were amplified (Supplemental Table S1), cloned into pGEM^®^-T Easy Vector System (Promega) and sequenced. A total of three colonies per clone were selected for sequencing.

### Reverse transcription semi-quantitative PCR (RT-qPCR) using total RNA

Total RNA was extracted from 80% confluent 6-well plates seeded initially with 1 × 10^5^ cells. Cells were then washed twice with sterile PBS and total RNA was extracted using the ReliaPrep RNA Cell Miniprep System (Promega, Dübendorf, Switzerland). RNA was eluted in 60 μl RNase free water and RNA concentration was measured using NanoDrop ND-1000 spectrophotometer (Thermo Fisher Scientific, Roskilde, Denmark).

Total RNA (500 ng) was reverse-transcribed using the iScript Reverse Transcription Supermix for RT-qPCR (Bio-Rad Laboratories, Zurich, Switzerland) in a total volume of 10 μl according to the manufacturer’s instructions. RT-qPCR was performed on target and reference genes (*HSPCB, SDHA*, and *YWHAZ*) in 10 μl reactions containing 10 ng cDNA (initial total RNA), 400 nM forward and reverse primer (Supplemental Table S2), nuclease free water and 1x GoTaq^®^ qPCR Master Mix with low ROX as the reference dye (Promega, Dübendorf, Switzerland) on a ViiA™ 7 Real-Time PCR System (Applied Biosystems, Thermo Fisher Scientific, Reinach, Switzerland). Quantitative PCR was performed on three independent experiments, in triplicates, and analyzed as recently described^19^,^46^. Each quantitative PCR was further established with a series of six 10-fold dilutions to calculate the efficacy of each reaction and to determine the optimal range of initial mRNA concentration for analysis (Supplemental Table S2).

### Flow cytometry

Immuno staining and flow cytometry was performed as previously described^14^,^15^. Biotinylated SNA lectin (1:500) containing 10 mM CaCl_2_ was applied for detection of α2-6 neuraminic acid using flow cytometry (BD Accuri™ C6, BD Bioscience).

### Membrane protein extraction and N- and O-glycan release of parental and ∆B3GNT5 IGROV1 cell lines

Membrane protein extraction and glycan release were carried out as previously described^19^. Briefly, approximately 2 × 10^7^ IGROV1 and ∆*B3GNT5* IGROV1 cells were washed with PBS and homogenized in lysis buffer (50 mM Tris-HCl, 100 mM NaCl, 1 mM EDTA and protease inhibitor, pH 7.4). Unlysed cells were pelleted through centrifugation and cellular membranes in the supernatant was subjected to ultracentrifugation at 120, 000 g prior to Triton X-114 phase partitioning of membrane proteins. Membrane proteins were acetone-precipitated, solubilized in 8 M urea and spotted onto polyvinylidene diflouride (PVDF) membrane spots placed in a 96-well microtiter plate. Protein spots were treated with PNG*ase* F enzyme and released *N*-glycans were treated with 100 mM ammonium acetate (pH 5.0) and reduced to alditols with 2 M NaBH_4_ in 50 mM KOH. For *O*-glycans, the remaining PVDF spots in the 96- well microtiter plate were further subjected to reductive β-elimination by treatment with 0.5 M NaBH_4_ in 50 mM KOH to chemically release the *O*-glycans. Both the released *N*- and *O*-glycans were desalted by cation exchange chromatography as previously described and re-suspended in 15 μl of MilliQ water prior to mass spectrometry analysis.

### GSL extraction and enzymatic release of GSL-glycans from cell lines

Lipid extraction and release of GSL-glycans were performed as previously described^13^. Briefly, GSLs were extracted from approximately 2 × 10^7^ parental and ∆*B3GNT5* IGROV1 cells using a modified Folch extraction method. Cell pellets were treated with chloroform: methanol (2:1) and subjected to centrifugation. The supernatants were evaporated to dryness and crude GSL fractions were re-dissolved in 50 μl of chloroform: methanol (2:1). Approximately 10 μl of GSL standard (neutral glycosphingolipids: LacCer, Gb3 and Gb4) and 50 μl of extracted GSLs were spotted onto the PVDF membrane and treated with 4 mU (2 μl) of Endoglycoceramidase II. GSL-glycans released from the immobilized GSL spots were reduced to alditols with 0.5 M NaBH_4_ in 50 mM KOH and desalted by cation exchange chromatography as previously described for *N*- and *O*- glycans.

### PGC-UHPLC-ESI-QTOF-MS/MS profiling of released GSL- and N-and O-glycan alditols

The separation of glycan alditols was performed using an Agilent UHPLC modular system consisting of a degasser, binary pump, auto-sampler, thermostat and column oven (Agilent Series 1290, Agilent Technologies, Germany). The conventional flow LC system was connected to a mass spectrometer (MS) (Agilent Series 6540 Q-TOF, Agilent Technologies, USA) operating in negative mode and coupled to a jet stream electrospray ion source (ESI) as previously described for *N*- and *O*-linked glycans^47^. Briefly with some slight modifications for GSL-derived glycans, an aliquot of 14 μL of sample was injected onto the Hypercarb porous graphitized carbon (PGC) analytical column (ID2.1 × 150 mm column; 3 μm particle size) (Thermo Fisher Scientific, United Kingdom). The column compartment was maintained at 40 °C. The mobile phase was made up of 10 mM ammonium bicarbonate (A) and 9:1 acetonitrile/water with 10 mM ammonium bicarbonate (B). The chromatographic separation was held with the initial mobile phase composition (1% B; 0–5 mins), followed by a segmented linear gradient to 25% B (5–40 mins), 50% B (40–42 mins) and a fast step at 42.5 mins to 80% B, with a hold for 1.5 mins. The ESI source was operated with the following parameter settings: nebulizer pressure (35 psig), nozzle voltage (0 V), sheath gas flow (11 L/min), sheath gas temperature (375 °C), drying gas flow (8 L/min), drying gas temperature (250 °C), capillary voltage (4000 V) and fragmentor voltage (175 V). High-resolution MS scan (*m*/*z* 100–1700 at 1.5 Hz) and MS/MS product scan (*m*/*z* 50–1700 at 3 Hz) were acquired with information-dependent acquisition (IDA) with static exclusion range *m*/*z* 100–300. An automated mass calibration performed by delivering constantly two reference masses (*m*/*z* 119.0363 and *m*/*z* 981.9956) on the end plate of the ESI source to obtain the highest mass accuracy during the measurement. Data analysis was performed with MassHunter B.06.00 (Agilent Technologies, USA). The monosaccharide compositions of monoisotopic masses were determined using GlycoMod tool (http://web.expasy.org/glycomod/) with a mass error of ±0.5 Da. The proposed glycan structures were manually assigned and interpreted based on MS/MS fragmentation patterns of known GSL-derived glycans^13^ and annotated using GlycoWorkBench 2.1 software^48^. In addition, UniCarb KB, a web-based LC-MS/MS database^49^, was also utilized to confirm fragmentation and retention time of sialylated *N*-glycans previously reported to contain sialylated isomers^19^. Blank samples, fetuin glycoprotein with isomeric glycan compositions and reference glycolipid standards which served as quality control for sample preparation were randomly measured within each sequence run and checked for background contamination.

### Confocal fluorescence microscopy

Cells were grown on polylysine glass slides attached to an 8-well chamber, fixed with 4% para-formaldehyde for 15 min and blocked with 5% (w/v) BSA fraction V (Sigma) dissolved in PBS for 1 h. Cells were then stained with either anti-P_1_ IgM^15^ (1:5 diluted with incubation buffer (1% BSA in PBS)) or anti-nLc4 (paragloboside) antibody (1:5) for overnight at 4 °C. Following extensive washing corresponding secondary antibodies (Biotin-anti-human IgM (1:100), Biotin- anti-mouse IgM (1:100)) were added to each chamber and incubated for 2 h at 4 °C. Afterwards, cells were washed with PBS and incubated with Streptavidin-FITC (1:200) for 1 h at 4 °C and counterstained with DAPI (Cell Signaling Technology). Fluorescence images were taken with a Zeiss LSM 710 confocal microscope (Zeiss, Feldbach, Switzerland).

### Printed Glycan Array

The printed glycan array was performed as previously described with modifications^29^,^50^,^51^. Briefly, glycan arrays were prepared by covalent attachment of amino-spacered synthetic glycans to *N*-hydroxysuccinimide-activated Schott-Nexterion slides. Chemically synthesized carbohydrates with 95–98% purity were used for printing and obtained from Lectinity Holdings (Moscow, Russia) and the Consortium for Functional Glycomics (CFG). The slides were washed for 15 min with 0.1 М PBS (0.01 М Na_2_HPO_4_, 0.01 M NaH_2_PO_4_, 0.138 M NaCl, and 0.0027 M KCl, pH 7.4) containing 0.1% Tween-20. Anti-P_1_ antibodies were diluted 1:50 in PBS containing 1% BSA. The slides with antibody solution were shaken for 1 h and incubated under relative humidity and temperature of 80% and 37 °C, respectively. The slides were washed with PBS and labeled with secondary antibodies consisting of goat anti-human IgM conjugated with Alexa647 (Invitrogen, USA), which was diluted with PBS (1:250). After incubation, the slides were washed with PBS containing 0.001% Tween 20 and subsequently with bi-distilled water and the fluorescence intensity was measured using a confocal ScanArray Gx scanner (PerkinElmer, USA) with 5 μm resolution. The obtained data were processed using ProScanArray Express 4.0 software.

### Proteinase K treatment

Proteinase K treatment was carried out as previously described^52^. Briefly, single-cell suspension of 2 × 10^6^ IGROV1, Ls174T, SKOV3 and *B3GNT5* - edited SKOV3 cells were treated with PBS containing 100 μg/mL proteinase K enzyme (Promega) for 1 h at 37 °C with intermittent shaking. At the same cell density the control cells were also resuspended in PBS without proteinase K. The enzymatic reaction was stopped by adding phenyl methyl sulfonyl fluoride (PMSF) at a concentration of 1 mM/mL and incubated on ice for 10 min. Cells were then fixed with 1% PFA on ice for 15 min and washed twice with 5 mL PBS and pelleted at 1200 rpm for 7 min at 4 °C. Finally, fixed cells were stained with SNA, nLc4 or anti-human CD44 antibody for flow cytometric analysis as previously described.

### Neuraminidase treatment

To remove the cell surface neuraminic acids, neuraminidase treatment was performed with α2,3-6, -8,- neuraminidase (New England BioLabs, UK) according to manufacturer’s instructions. In brief, IGROV1 cells (1 × 10^6^) were fixed with 4% paraformaldehyde in PBS at room temperature for 15 min, and then washed with PBS twice. Cells were resuspended in 200 μl of glycobuffer (1x) containing 25 Units of the enzyme and incubated at 37 °C for 2 h. Cells were washed with PBS and followed by SNA staining and flow cytometry.

### Statistical analysis

Experiments were performed in triplicates and comparisons were statistically evaluated with two-tailed Student’s *t*-test. *P* values of <0.05 were considered statistically significant (***p < 0.001, **p < 0.01, *p-value < 0.05, *p*-value < 0.1.).

## Additional Information

**How to cite this article:** Alam, S. *et al*. Altered (neo-) lacto series glycolipid biosynthesis impairs a2-6 sialylation on *N*-glycoproteins in ovarian cancer cells. *Sci. Rep.* **7**, 45367; doi: 10.1038/srep45367 (2017).

**Publisher's note:** Springer Nature remains neutral with regard to jurisdictional claims in published maps and institutional affiliations.

## Supplementary Material

Supplementary Information

## Figures and Tables

**Table 1:** Proposed GSL-glycan structures detected on the glycolipid membranes of ovarian cancer cells, IGROV1 (wildtype) and *∆B3GNT5* cells.

Type	No	Glycan Mass [M-H]^-^	Glycan Structures	PGC-ESI-QTOF-MS/MS		PGC-ESI-IT-MS/MS
				IGROV1	B3GNT5-edited IGROV1	P_1_-enriched IGROV1 (Anugraham M, *et al*, 2015)
**Neutral GSL**	1	505.2 (Pk)	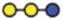	6.34 ± 0.31	4.72 ± 2.38	0.00
	2	546.3	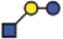	0.00	0.00	0.00
	3a	708.3 (Globo)	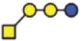	0.00	0.00	0.00
	3b	708.3 (Asialo-GM1)	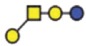	0.00	0.00	0.00
	3c	708.3 (Neo-lacto)	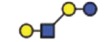	4.52 ± 1.58	0.00	7.64
	4	870.3 (P1)	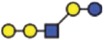	0.00	0.00	2.43
	5	911.3	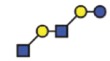	1.25 ± 0.48	0.00	Trace
**Sialylated GSL**	6	634.2 (GM3)	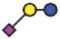	14.18 ± 0.61	23.40 ± 4.32	12.86
	7	837.3 (GM2)	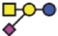	73.71 ± 2.40	71.88 ± 8.13	73.11
	8a	999.3 (GM1)	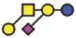	0.00	0.00	0.68
	8b	999.3 (α2-3 sialyl Paragloboside)	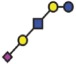	0.00	0.00	2.15
	8c	999.3 (LSTc)	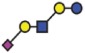	0.00	0.00	1.14

Structures were assigned based on MS/MS fragmentation (where possible) and known biological GSL synthetic pathway constraints. All structures were depicted according to the CFG (Consortium of Functional Glycomics) notation with linkage placement. Specific linkages corresponding to Gal-GlcNAc (Type 1/Type 2) lactosamine linkages are also indicated (where possible). Values represent mean relative ion intensities ± s.d. of three replicates based on their extracted ion chromatograms (EIC). GSL-glycan structures derived from P_1_-enriched IGROV1 cell line previously analyzed with PGC-ESI-IT-MS (20) are also shown for comparative profiling using two different MS platforms. Gb3 (Pk).

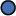
 Glucose 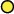 Galactose 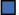
*N*-acetylglucosamine (GlcNAc) 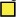
*N*-acetylgalactosamine (GalNAc) 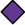
*N*-acetylneruaminic acid (NeuAc)

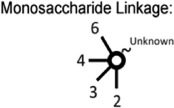

**Table 2:** Proposed *N*- and *O*-glycan structures detected on the membrane proteins of IGROV1 (wildtype) and *∆B3GNT5* cells.

Type	No	Glycan Mass [M-H]^−^	[M-2H]^2−^	Glycan Structures	PGC-ESI-QTOF-MS/MS		PGC-ESI-IT-MS/MS
					IGROV1	B3GNT5-edited IGROV1	IGROV1 (Anugraham M, *et al*, 2014)
***N*** **-glycans -High Mannose**	1	1235.4	617.2	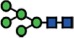	4.15 ± 0.11	5.54 ± 0.16	0.62 ± 0.06
	2	1397.6	698.3	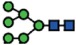	5.31 ± 0.24	4.88 ± 0.64	7.91 ± 0.15
	3	1559.6	779.3	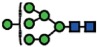	9.13 ± 0.27	9.48 ± 1.21	13.45 ± 1.35
	4	1721.6	860.3	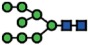	19.19 ± 1.15	20.85 ± 1.57	16.00 ± 3.73
	5	1883.8	941.4	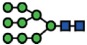	23.48 ± 2.02	22.29 ± 0.79	24.57 ± 6.02
	6	2045.6	1022.3	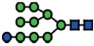	1.88 ± 0.21	1.73 ± 0.52	1.43 ± 0.61
***N*** **-glycans -Hybrid**	7	1567.6	783.3	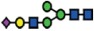	0.00	0.00	0.19 ± 0.03
	8	1584.6	791.8	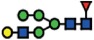	0.00	0.00	0.34 ± 0.11
	9	1600.6	799.8	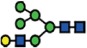	0.63 ± 0.01	0.83 ± 0.12	1.04 ± 0.30
	10	1713.6	856.3	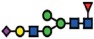	0.00	0.00	1.76 ± 0.21
	11	1729.6	864.3	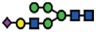	0.00	0.00	0.68 ± 0.20
	12	1746.6	872.8	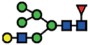	0.00	0.00	0.20 ± 0.00
	13a	1875.6	937.3 (α2-6)	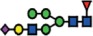	0.65 ± 0.03	0.00	0.20 ± 0.25
	13b	1875.6	937.3 (α2-3)	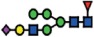	0.42 ± 0.05	0.74 ± 0.07	0.41 ± 0.20
	14a	1891.6	945.3 (α2-6)	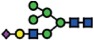	1.34 ± 0.09	0.00	0.47 ± 0.22
	14b	1891.6	945.3 (α2-3)	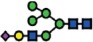	0.67 ± 0.05	1.33 ± 0.17	0.43 ± 0.08
***N*** **-glycans -Complex Neutral Hybrid**	15	1463.6	731.2	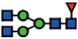	1.25 ± 0.20	1.36 ± 0.35	0.74 ± 0.03
	16	1625.6	812.3	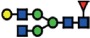	0.00	0.14 ± 0.25	0.64 ± 0.14
	17	1641.6	820.3	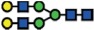	0.62 ± 0.01	1.16 ± 0.18	1.59 ± 0.62
	18	1666.4	832.8	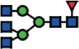	0.00	0.00	1.22 ± 0.40
	19	1682.6	840.8	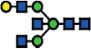	0.00	0.00	0.12 ± 0.05
	20	1771.8	885.4	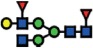	0.00	0.00	0.00
	21	1787.6	893.3	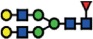	1.99 ± 0.05	3.48 ± 0.47	3.50 ± 0.22
	22	1812.8	905.9	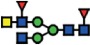	0.00	0.00	0.24 ± 0.08
	23	1828.8	913.9	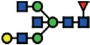	0.00	1.30 ± 0.34	0.66 ± 0.12
	24	1844.8	921.9	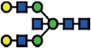	1.25 ± 0.06	2.01 ± 0.09	1.37 ± 0.19
	25	1869.8	934.4	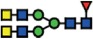	0.00	0.19 ± 0.33	0.51 ± 0.20
	26	1933.6	966.3	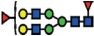	1.23 ± 0.78	1.15 ± 0.13	0.00
	27	1974.8	986.9	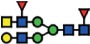	0.18 ± 0.31	0.00	0.79 ± 0.28
	28	1990.8	994.9	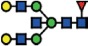	6.29 ± 1.36	8.23 ± 0.73	2.62 ± 0.60
	29	2006.8	1002.9	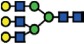	1.84 ± 0.39	1.75 ± 0.61	0.31 ± 0.15
	30	2015.8	1007.4	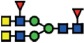	0.00	0.00	0.14 ± 0.06
	31	2079.8	1039.4	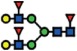	0.00	0.00	0.00
	32	2120.8	1059.9	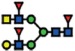	0.00	0.00	0.56 ± 0.19
	33	2136.6	1067.8	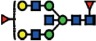	2.32 ± 0.27	3.44 ± 0.35	0.31 ± 0.15
	34	2152.8	1075.9	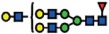	0.77 ± 0.40	1.47 ± 0.95	1.18 ± 0.16
	35	2162	1080.5	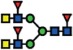	1.22 ± 0.22	0.25 ± 0.15	0.39 ± 0.09
	36	2356	1177.5	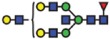	0.84 ± 0.07	1.13 ± 0.14	0.23 ± 0.02
	37	2518	1258.5	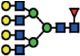	1.88 ± 0.24	1.20 ± 0.22	0.37 ± 0.10
***N*** **-glycans -Complex Sialylated Hybrid Complex Sialylated**	38	1916.6	957.8	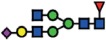	0.00	0.00	0.21 ± 0.06
	39a	1932.8	965.9 (α2-6)	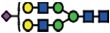	1.41 ± 0.11	0.00	1.38 ± 0.38
	39b	1932.8	965.9 (α2-3)	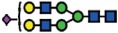	0.86 ± 0.05	1.28 + 0.24	1.86 ± 0.49
	40a	2078.8	1038.9 (α2-6)	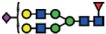	4.96 ± 0.35	0.00	1.91 ± 0.23
	40b	2078.8	1038.9 (α2-3)	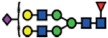	1.68 ± 0.16	3.94 ± 0.27	3.23 ± 0.14
	41	2119.8	1059.4	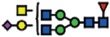	0.00	0.00	0.36 ± 0.15
	42	2223.8	1111.4	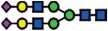	0.00	0.00	0.41 ± 0.07
	43	2265.8	1132.4	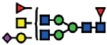	0.00	0.00	0.06 ± 0.02
	44a	2282.0	1140.5 (α2-6)	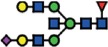	1.35 ± 0.11	0.00	0.35 ± 0.14
	44b	2282.0	1140.5 (α2-3)	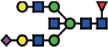	1.20 ± 0.34	2.30 ± 0.06	0.30 ± 0.17
	45	2297.8	1148.4	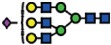	0.00	0.00	0.23 + 0.02
	46	2370.0	1184.5	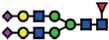	0.00	0.00	1.33 ± 0.36
	47	2411.0	1205	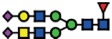	0.00	0.00	0.06 ± 0.02
	48	2444.0	1221.5	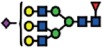	1.30 + 0.46	0.00	0.65 ± 0.07
	49	2589.0	1294.0	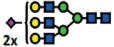	0.00	0.00	0.03 ± 0.00
	50	2735.0	1367.0	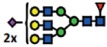	0.00	0.00	0.03 ± 0.00
	51	3026.0	1512.5	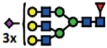	0.00	0.00	0.19 ± 0.08
**O-glycans**	1a	675.3	NA	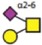	9.10 ± 2.32	13.08 ± 2.43	NA
	1b	675.3	NA	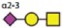	31.55 ± 4.19	27.59 ± 2.21	NA
	2	966.3	NA	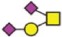	52.11 ± 2.60	52.25 ± 0.59	NA
	3	749.3	NA	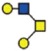	0.00	0.00	NA
	4	1040.5	NA	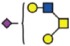	7.24 ± 1.26	7.08 ± 0.44	NA
	5	1331.5	NA	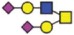	0.00	0.00	NA

*N*- and *O*-glycan structures were separated by PGC-UHPLC-ESI-QTOF and their structures were assigned based on MS/MS fragmentation (where possible), retention time differences and biological pathway constraints. Structures were depicted according to the Consortium of Functional Glycomics (CFG) notation with linkages (α2, 3 and α2, 6) indicated for sialic acid (where known). Values represent mean relative ion intensities ± SD of three replicates based on their extracted ion chromatograms (EIC). *N*-glycan structures derived from IGROV1 cell line previously analyzed with PGC-ESI-IT-MS^13^,^19^ are also shown for comparative profiling using two different MS platforms.

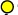
 Galactose 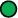 Mannose 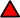 Fucose 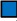
*N*-acetylglucosamine (GlcNAc) 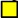
*N*-acetylgalactosamine(GalNAc) 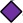
*N*-acetylneruaminic acid (NeuAc; sialic acid).
