# Supplementary material for: Altered (neo-) lacto series glycolipid biosynthesis impairs α2-6 sialylation on N-glycoproteins in ovarian cancer cells
Source: Sci Rep. 2017 Mar 30;7:45367. doi: 10.1038/srep45367 (PMC5371825; doi:10.1038/srep45367)
Supplement: Supplementary Information [file srep45367-s1.pdf]

# Altered (neo-) lacto series glycolipid biosynthesis impairs α2-6 sialylation on *N*-glycoproteins in ovarian cancer cells

Shahidul Alam[1,2+], Merrina Anugraham[1+], Yen-Lin Huang[1], Reto S. Kohler[1], Timm Hettich[3], Katharina Winkelbach[1], Yasmin Grether[1], Mónica Núñez López[1], Nailia Khasbiullina[4], Nicolai V. Bovin[4], Götz Schlotterbeck[3], Francis Jacob[1,2*]

**Supplementary information**

**Supplemental Fig. S1**

**Single- cell selection after transient transfection of CRISPR- *Cas9* encoding plasmids.** Representative gating of fluorescence activated single cell sorting into 96-well plates after transient delivery of pSpCas9(BB)-2A-GFP (PX458) encoding sgRNA.

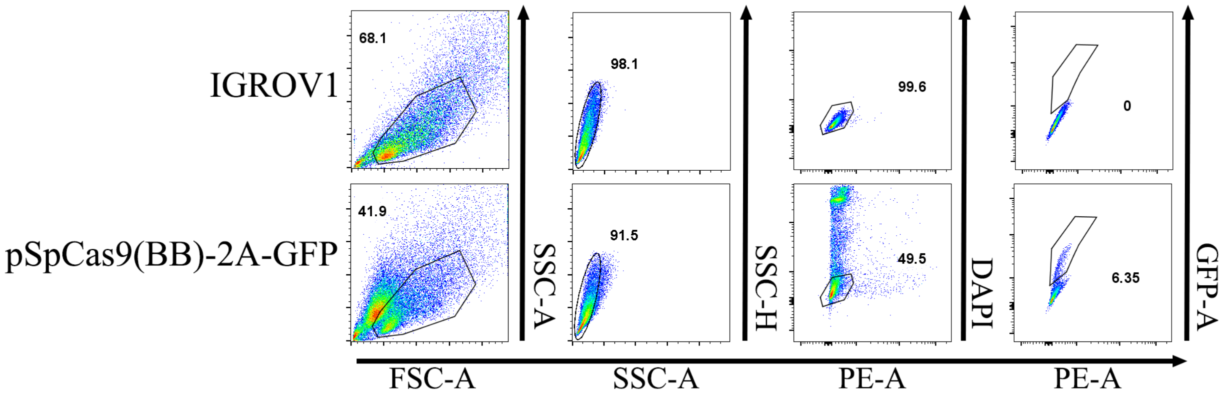

**Supplemental Fig. S2**

**Determination and verification of potential off targets.** *In silico* analysis revealed 50 and 17 potential off-targets for sgRNA_1 and sgRNA_2, respectively. The sgRNA_1 had only one predicted off-target in gene NM_001242377 chr5:+112339780 whereas sgRNA_2 was not predicted for an off-target site in a gene. Genomic sequences of predicted off-targets with highest score for each sgRNA were further amplified and sequenced, in which no mutations were observed. Off target DNA sequencing results for B3GNT5 wildtype (IGROV1) and knockout (clone KO_1 and KO_2) cells performed in triplicates. Genomic DNA sequence according to February 2009 human reference sequence (http://genome.ucsc.edu/index.html, GRCh37). Standard SP6 DNA Sequencing primer was used.

```
Off target 5'- ata cac aat tac agt ctt ttc aga aat tta gat -3'       GENOME

01_SP6_    5'- ata cac aat tac agt ctt ttc aga aat tta gat -3'  OK   clone KO_1
02_SP6_    5'- ata cac aat tac agt ctt ttc aga aat tta gat -3'  OK   clone KO_1
03_SP6_    5'- ata cac aat tac agt ctt ttc aga aat tta gat -3'  OK   clone KO_1

04_SP6_    5'- ata cac aat tac agt ctt ttc aga aat tta gat -3'  OK   clone KO_2
05_SP6_    5'- ata cac aat tac agt ctt ttc aga aat tta gat -3'  OK   clone KO_2
06_SP6_    5'- ata cac aat tac agt ctt ttc aga aat tta gat -3'  OK   clone KO_2

07_SP6_    5'- ata cac aat tac agt ctt ttc aga aat tta gat -3'  OK   IGROV1
08_SP6_    5'- ata cac aat tac agt ctt ttc aga aat tta gat -3'  OK   IGROV1
09_SP6_    5'- ata cac aat tac agt ctt ttc aga aat tta gat -3'  OK   IGROV1
```

**Supplemental Fig. S3**

**Determination of anti- $P_1$ IgM binding epitopes using printed glycan array.** Barchart demonstrating the binding specificity for human anti-$P_1$ IgM P3NIL100 antibodies. We performed a broad screening of glycan binding specificity of the monoclonal antibody P3NIL100 across 381 glycans by utilizing glycan array technology. The results clearly demonstrated that antibody bound specifically to glycans with Galα1-4Galβ1-4GlcNAcβ as well as glycans terminating with Galα1-3(Galα1-4)Galβ1-4 and GalNAcα1-4Galβ1-4, with some of these structures bearing α1-2-linked fucose. As expected, there was no binding of the antibody to Gb3 glycan (Galα1-4Galβ1-4Glcβ-sp).

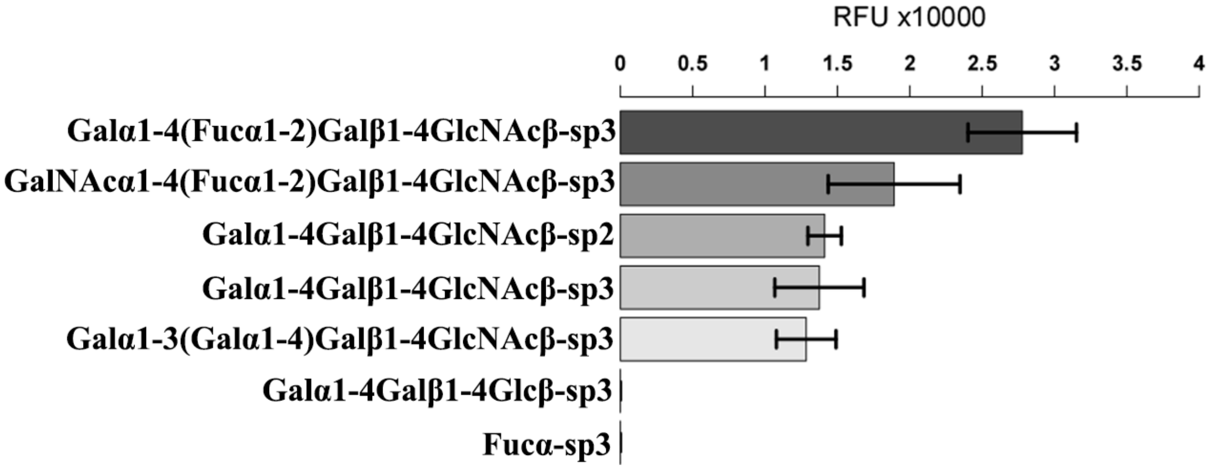

**Supplemental Fig. S4**

**The epitope for anti- nLc4 IgM is present on glycoproteins in colon cancer cell line Ls174T. A)** Histogram showing the absence of α2-6 sialylation in Ls174T cells. **B)** Proteinase K treatment on Ls174T cells following staining for CD44 and nLc4. Representative histogram for unstained (white), Proteinase K -untreated (dark gray) and -treated cells (light gray). Data are represented as mean ± s.d, *** $p < 0.001$, ** $p < 0.01$.

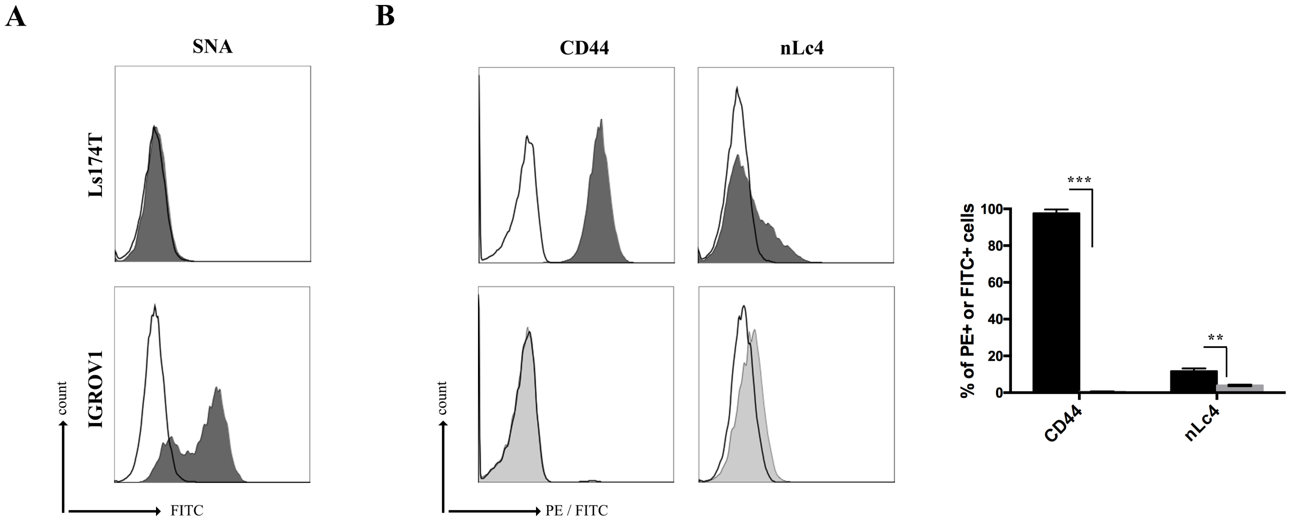

**Supplemental Fig. S5**

**Quantitative gene expression of *ST6GAL1*, *B4GALNT1*, and *B3GNT5* in IGROV1, Δ*B3GNT5-, and* Δ*B4GALNT1-* cells.** Barchart represents three independent experiments. Data are represented as mean ± s.d. RT-qPCR was performed in triplicates on three independent experiments.

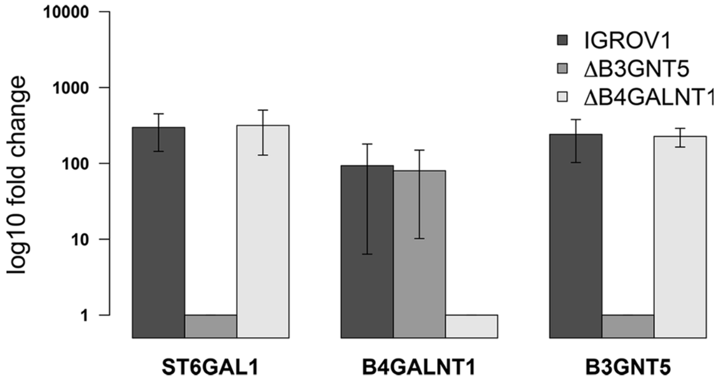

## Supplemental Table S1

Oligonucleotides used in the study.

| | | | | |
|---|---|---|---|---|
| **single guided RNAs** | | | | |
| | oligonulceotide 5 '- 3' | | Gene | Method |
| sgRNA 1 | CAC CGA TAC ACG ATT ATA GCC GTT T | AAA CAA ACG GCT ATA ATC GTG TAT C | B3GNT5 | Gene-editing |
| sgRNA 2 | CAC CGA TCG ACG TTC CGG AAT TAG A | AAA CTC TAA TTC CGG AAC GTC GAT C | B3GNT5 | Gene-editing |
| sgRNA 1 | CAC CGT GGA TGC CGC GGT TTC GAC G | AAA CCG TCG AAA CCG CGG CAT CCA C | B4GALNT1 | Gene-editing |
| sgRNA 2 | CAA CGC CTT CAA ATA GTC CTC GGG A | AAA CTC CCG AGG ACT ATT TGA AGG C | B4GALNT1 | Gene-editing |
| | | | | |
| **genotyping PCR primer** | | | | |
| | oligonulceotide 5 '- 3' (Forward) | oligonulceotide 5 '- 3' (Reverse) | Gene | Method |
| PCR_1 | GTA TCT GCT TTC ATC CTG ACC AT | TGC CCA ACT GAA CTG CAT AAG | B3GNT5 | Genotyping |
| PCR_2 | GTA TCT GCT TTC ATC CTG ACC AT | TCG AGA CCA TAG AAC TTC GTG T | B3GNT5 | Genotyping |
| PCR_3 | GGC CTC GCT ACC AAT ACT T | TGC CCA ACT GAA CTG CAT AAG | B3GNT5 | Genotyping |
| PCR_4 | GAT GAG GAT AAA GCA GTG CAG AT | GCC TCA GCC CTG CTT CAA ATC | chr5:113003768+113004235 | off target |
| | | | | |
| PCR_1 | AGA GCG TTA GAC AGG TCA GT | TGG AGG AAG GAG AGG ACA GA | B4GALNT1 | Genotyping |
| PCR_2 | CTG TGC GCT CTG GTC CTT | TGG AGG AAG GAG AGG ACA GA | B4GALNT1 | Genotyping |
| PCR_3 | CCA CTA CTT GCT CCT TGA TCC | TGG AGG AAG GAG AGG ACA GA | B4GALNT1 | Genotyping |
| PCR_4 | CCA CTA CTT GCT CCT TGA TCC | CTG TGC GCT CTG GTC CTT | B4GALNT1 | Genotyping |
| PCR_5 | GCA CAT ACT GAA GCG TTC ACA | ATC CCA GCT CCT CAG CTA CA | chr22:30296425-30296628 | off target |
| PCR_6 | TTG GCC AGA GGT TCA TGC TA | ACC GTC ACC CTA CTC AAG TG | chr12:52518025-52518321 | off target |

**Supplemental Table S2**

Details of target and reference genes, primers and amplicons investigated in this study by RT-qPCR. QPCR parameters are provided including efficiency (E in %; calculated based on the standard curve according to the equation E=$10^{(-1/slope)}$-1) x 100 and expressed as percentage) and correlation coefficient ($R^2$).

| Symbol | Gene name | Accession number | Chr. location | Forward Primer 5‘-3‘ | Reverse Primer 5‘-3 | Amplicon size in bp | E in % | $R^2$ |
|---|---|---|---|---|---|---|---|---|
| ***B3GNT5*** | UDP-GlcNAc β -1,3-*N*-acetylglucosaminyl-transferase 5 | NM_032047.4 | 3q28 | GGCTTGAACTTCGT GAGTTTCGC | TCGAGACCATAGAA CTTCGTGT | 305 | 92.2 | 0.994 |
| ***ST6GALNAC1*** | GalNAc α -2,6-Sialyltransferase I | NM_018414.3 | 17q25.1 | CGAAATAGGAGGCC TTCAGACGAC | TTTCTGGAGCCACA GCGACTTG | 76 | 74.1 | 0.999 |
| ***ST6GALNAC2*** | GalNAc α -2,6-Sialyltransferase II | NM_006456.2 | 1q31.1 | ATCGAATTCTTGGA CAGGAAAGGG | GATTGAACAGGCCA CGGAAGTG | 92 | 83.9 | 0.899 |
| ***ST6GALNAC3*** | GalNAc α -2,6-Sialyltransferase III | NM_001160011.1 | 1q31.1 | AGCGCATGAGTTAC TGTGATGG | TGCATGGTCACTCT GTACTGTCC | 69 | 103.9 | 0.999 |
| ***ST6GALNAC4*** | GalNAc α -2,6-Sialyltransferase IV | NM_175039.3 | 9q34.11 | ACCAGATCTTCCAG GACGAGAC | TCTTCTCCCTGCAGT AGCTGTC | 150 | 86.7 | 0.998 |
| ***ST6GALNAC5*** | GalNAc α -2,6-Sialyltransferase V | NM_030965.1 | 1q31.1 | TGACAATTGCACTG GAGCTCTG | GTGATTGGGATCCC TGCAGAAG | 80 | 131.6 | 0.955 |
| ***ST6GALNAC6*** | GalNAc α -2,6-Sialyltransferase VI | NM_013443.3 | 9q34.11 | CATGCGGCAATTTG ACGACCTC | CACGAATGAGACTT CTCCCTGTCC | 66 | 106.1 | 0.999 |
| ***ST6GAL1*** | β -galactosamide α-2,6-sialyltransferase 1 | NM_173217.2 | 3q27-q28b | CCATCCTCTGGGAT GCTTGGTATC | ACGTCAGTCTTGCG CTTGGATG | 95 | 98.1 | 0.997 |
| ***ST6GAL2*** | β -galactosamide α-2,6-sialyltransferase 2 | NM_001142351.1 | 2q12.3 | TTCCTTGGGCGAGG AAATAGATTC | TCATAACCACGTGT AGGAGCAGAG | 72 | 99.2 | 0.991 |
| ***B4GALNT1*** | β -1,4-*N*-Acetyl-Galactosaminyl Transferase 1 | NM_001276468 | 12q13.3 | TGTGCGCTCTGGTCC TTCTG | TTGATCCTGACCGG GATGTGT | 181 | 74.1 | 0.999 |
| ***HSPCB*** | Heat shock protein 90kDa alpha (cytosolic) | NM_007355 | 6p12 | TCTGGGTATCGGAA AGCAAGCC | GTGCACTTCCTCAG GCATCTTG | 80 | 87.2 | 0.999 |
| ***YWHAZ*** | Tyrosine 3-monooxygenase/ tryptophan 5-monooxygenase activation protein | NM_003406 | 8q23.1 | ACTTTTGGTACATTG TGGCTTCAA | CCGCCAGGACAAAC CAGTAT | 94 | 94.1 | 0.999 |
| ***SDHA*** | Succinate dehydrogenase complex, subunit A | NM_004168 | 5p15 | TGGGAACAAGAGGG CATCTG | CCACCACTGCATCA AATTCATG | 86 | 100.9 | 0.999 |
